# Linking the Planetary Health Diet Index to sarcopenia: the mediating effect of the non-high-density lipoprotein cholesterol to high-density lipoprotein cholesterol ratio (NHHR)

**DOI:** 10.3389/fnut.2025.1504037

**Published:** 2025-06-19

**Authors:** Huan Chen, Zhao Wang, Changsin Lee, Rongcan Liu, Junlong Song, Junsheng Zhang, Ning Du, Chan Kang

**Affiliations:** ^1^Department of Orthopedic Surgery, Chungnam National University School of Medicine, Daejeon, Republic of Korea; ^2^Department of Cosmetic Dermatology, University-Town Hospital of Chongqing Medical University, Chongqing, China; ^3^Department of Orthopedic of Jiangbei Campus, The First Affiliated Hospital of Army Medical University, Chongqing, China; ^4^Department of Psychiatry, University-Town Hospital of Chongqing Medical University, Chongqing, China

**Keywords:** PHDI, NHHR, sarcopenia, NHANES, mediation analysis

## Abstract

**Background:**

The Planetary Health Diet Index (PHDI), which promotes plant-based food consumption and limits red meat and processed food intake, aligns with goals for human health and environmental sustainability. Sarcopenia, characterized by progressive muscle loss, has been increasingly associated with dietary and metabolic factors. This study aims to explore the relationship between PHDI and sarcopenia and investigates the potential mediating role of the non-high-density lipoprotein to high-density lipoprotein cholesterol ratio (NHHR).

**Methods:**

A total of 9,094 individuals from the National Health and Nutrition Examination Survey (NHANES), conducted between 2011 and 2018, were included in this analysis. Multivariable logistic regression, smooth curve fitting, and subgroup analyses were applied to explore the association between the PHDI and the likelihood of sarcopenia. Additionally, mediation analysis was performed to assess the potential mediating role of NHHR.

**Results:**

The findings indicated a notable negative association between PHDI and sarcopenia. After adjusting for confounding factors, a 10-unit increase in PHDI was associated with an 14% lower likelihood of sarcopenia [Odds Ratio (OR) = 0.86, 95% Confidence Interval (CI): 0.79, 0.94]. Participants in the highest PHDI tertile (T3) were observed to have a 38% reduced likelihood of sarcopenia compared to those in the lowest tertile (T1) [OR = 0.62, 95% CI: 0.43, 0.90]. Analysis of the dose–response curve suggested a linear relationship between PHDI and sarcopenia. Furthermore, a significant positive association was identified between NHHR and sarcopenia [OR = 1.09, 95% CI: 1.03, 1.16], with NHHR found to decrease as PHDI increased [beta coefficient (*β*) = −0.09, 95% CI: −0.11, −0.06]. Mediation analysis revealed that NHHR partially mediated the relationship between PHDI and sarcopenia, accounting for 8.33% of the total effect.

**Conclusion:**

This study highlights the observed negative correlation between PHDI and sarcopenia, with NHHR acting as a partial mediator. These findings emphasize the potential importance of dietary patterns in strategies aimed at preventing sarcopenia.

## Introduction

Sarcopenia, a prevalent degenerative muscle condition predominantly affecting older individuals, has emerged as a critical global public health concern. It is characterized by a marked reduction in skeletal muscle mass and strength, leading to diminished mobility, increased risk of falls, and reduced quality of life (1). Currently, approximately 50 million people worldwide are estimated to have sarcopenia, with this number projected to rise to 200 million by 2050 (2). The global prevalence ranges between 10 and 30%, with an anticipated increase as populations continue to age (3). Although prevalence rates vary by region, they are notably higher in developed countries and areas experiencing rapid demographic changes (4). Sarcopenia significantly contributes to disability, functional decline, and rising healthcare costs among the elderly (5). Its impact not only reduces the quality of life for affected individuals but also places a considerable strain on global healthcare systems, exacerbating the burden of non-communicable diseases (6).

Unhealthy eating habits are major contributors to chronic conditions such as obesity, cardiovascular diseases, diabetes, and premature mortality. These habits also adversely affect the environment, contributing to greenhouse gas emissions, resource depletion, and biodiversity loss (7, 8). The EAT-Lancet Commission introduced the Planetary Health Diet Index (PHDI) in 2019 to address these interlinked challenges (9). PHDI emphasizes plant-based dietary patterns, encouraging the consumption of whole grains, vegetables, fruits, legumes, and nuts while limiting red meat, sugar, and processed foods. This diet aims to enhance public health by reducing chronic disease risks and simultaneously promoting environmental sustainability. However, evidence regarding the relationship between PHDI and sarcopenia remains an unexplored area.

Growing evidence underscores a complex link between obesity and sarcopenia, with obesity contributing to insulin resistance, chronic inflammation, disrupted glucose metabolism, and dyslipidemia (10, 11). Non-high-density lipoprotein to high-density lipoprotein cholesterol ratio (NHHR), reflecting the balance between pro-and anti-atherogenic lipids, has emerged as a promising marker for assessing lipid imbalances in conditions like atherosclerosis and insulin resistance (12). Recent studies suggest NHHR is associated with sarcopenia (13, 14). However, its potential role as a mediator in the relationship between PHDI adherence and sarcopenia remains underexplored.

This study aims to address this gap by investigating the association between adherence to the PHDI and sarcopenia using a comprehensive cross-sectional analysis of National Health and Nutrition Examination Survey (NHANES) data. We hypothesize that higher adherence to a healthy dietary pattern, as reflected by a higher PHDI, is inversely associated with sarcopenia. Furthermore, we propose that the NHHR mediates this relationship. Specifically, we posit that healthier dietary patterns improve lipid metabolism by lowering NHHR, thereby reducing pro-inflammatory processes that contribute to muscle degradation. By exploring these mechanisms, our study seeks to offer novel insights into the interplay between diet, lipid metabolism, and sarcopenia, with significant implications for targeted prevention and management strategies.

## Methods

### Study population

**Figure 1:**
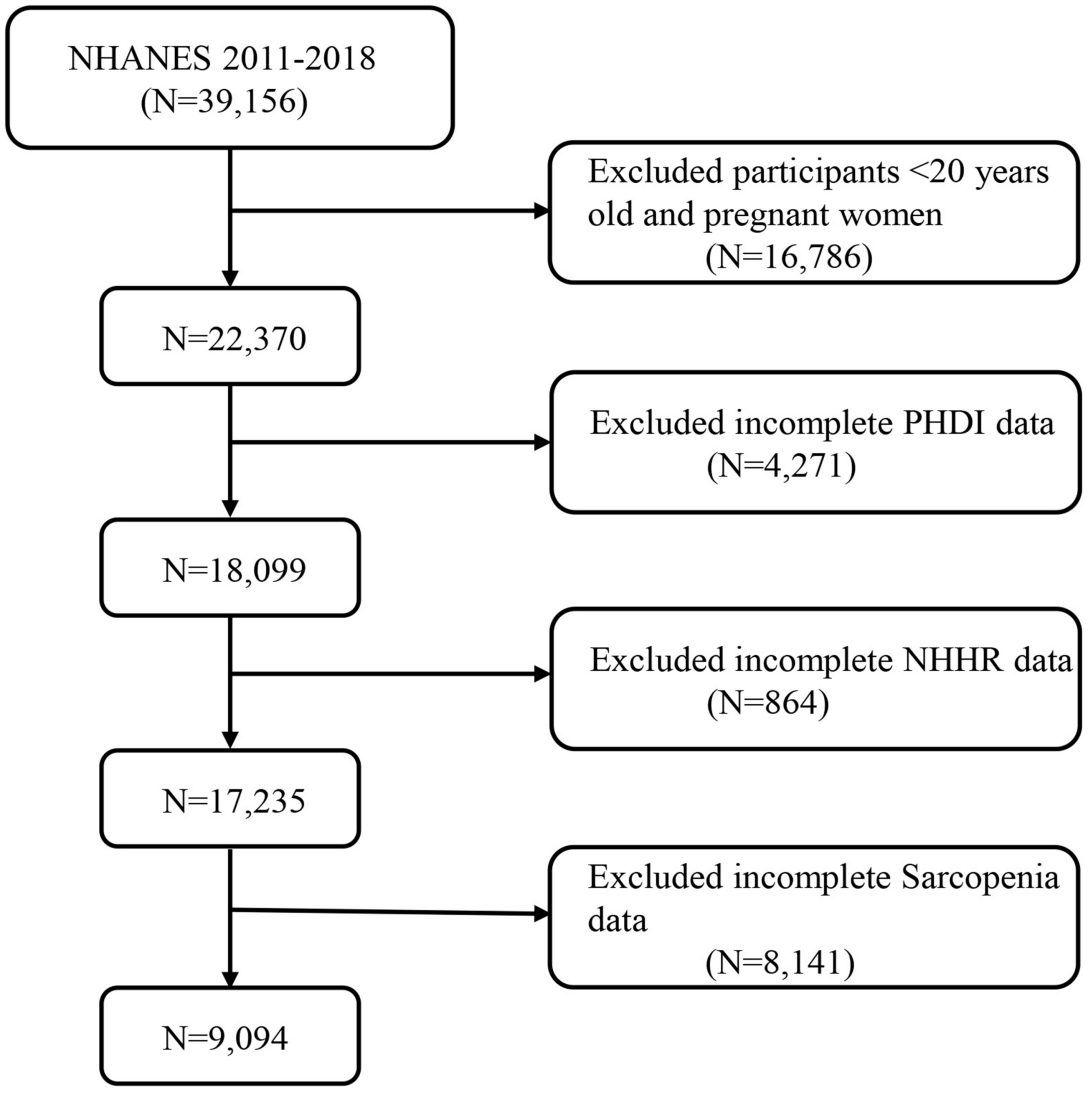
A flow diagram of eligible participant selection in the National Health and Nutrition Examination Survey. PHDI, Planetary Health Diet Index; NHHR, non-high-density lipoprotein cholesterol to high-density lipoprotein cholesterol ratio.

The NHANES is designed as a comprehensive cross-sectional investigation conducted across the U. S., aimed at assessing the health and nutritional status of the populace. Data are gathered via household interviews along with clinical assessments performed at Mobile Examination Centers (MECs). Ethical approval for NHANES was obtained from the Ethics Review Board of the National Center for Health Statistics (NCHS), with all participants providing informed consent. For this analysis, we drew from publicly accessible, anonymized data from NHANES cycles spanning 2011 to 2018, encompassing 39,156 participants. Participants in our study ranged in age from 20 to 85 years. We omitted individuals under 20 years of age and pregnant women (*n* = 16,786), in addition to participants with incomplete or unavailable PHDI data (*n* = 4,271), those missing NHHR data (*n* = 864), and individuals without sarcopenia-related details (*n* = 8,141). Following these exclusions, our final analysis included 9,094 participants, with the comprehensive selection process illustrated in Figure 1.

### Definition of PHDI and NHHR

The key variable under investigation in this research was the PHDI, which was calculated following the methodology established by the EAT-Lancet Commission. The PHDI score varies between 0 and 140 and includes 14 unique food categories organized into two primary groups. The first group represents foods that should be consumed in higher amounts, such as whole grains, fruits, vegetables, legumes, nuts, seeds, and unsaturated fats. In contrast, the second group pertains to foods that should be limited and includes red meat, processed meat, dairy products, poultry, eggs, fish, saturated fats, and added sugars. A score from 0 to 10 was assigned to each food category based on the levels of consumption. For each food group, scores were assigned proportionally based on the intake levels between the minimum and maximum thresholds (15). More detailed information regarding the PHDI scoring methodology, along with additional studies and Supplementary Table S1, can be found in references (16, 17).

The NHHR is determined by dividing non-High Density Lipoprotein (HDL) cholesterol—calculated as total cholesterol subtracting HDL cholesterol—by HDL cholesterol itself. Generally, this measurement is obtained from lipid profiles taken while fasting (18).

### Definition of sarcopenia

The measurement of appendicular skeletal muscle mass (ASM) was conducted through dual-energy X-ray absorptiometry (DXA). According to the standards set by the Foundation for the National Institutes of Health (FNIH), sarcopenia is defined based on ASM that is adjusted for body mass index (BMI). In particular, sarcopenia is diagnosed when the ASM/BMI ratio falls below 0.512 for women and below 0.789 for men (19). Participants exceeding DXA operational limits, including those who were pregnant, weighed over 136 kg, or were taller than 196 cm, were excluded to ensure safety and measurement accuracy.

### Covariates

The analysis included a comprehensive range of covariates informed by previous research, such as age, gender, education level, marital, poverty income ratio (PIR), race, smoking, drinking, hypertension, diabetes (a history of diabetes primarily refers to type 2 diabetes, the predominant form in the study population, with limited inclusion of type 1 cases, which were not analyzed separately), total energy intake, protein intake, Urea level, and total serum protein. Further details regarding these covariates can be found in the Supplementary Table S2.

### Statistical analysis

Statistical analyses were performed using R software (version 4.3.1). To guarantee that the data accurately represented the U. S. population, we applied weights recommended by NHANES. In particular, the two-day dietary sample weight (WTDR2D) was utilized, with the weight for the years 2011–2018 computed as 1/4 × WTDR2D. Continuous variables are reported as mean ± standard deviation (SD), whereas categorical variables are shown as percentages (20). To compare continuous variables, *t*-tests were conducted, and chi-square tests were used to evaluate differences between individuals who are sarcopenic and those who are not.

Tertiles for both PHDI and NHHR were constructed based on the 33rd and 66th percentiles, calculated using survey-weighted quantiles from the NHANES design. The lowest tertile was defined as the reference group.

To investigate the relationships involving the PHDI, NHHR and sarcopenia, we applied weighted multivariable logistic regression analyses. We developed three distinct models to adjust for confounding variables: (1) a model without adjustments, (2) a model modified for factors such as age, gender, educational attainment, marital status, PIR, and ethnicity, and (3) a comprehensive model that also incorporated variables like smoking status, alcohol consumption, hypertension, diabetes, total energy intake, protein intake, Urea level, and total serum protein. The Odds Ratio (OR) for the associations between PHDI, NHHR, and sarcopenia were calculated per 10-unit increase. In addition, we utilized weighted linear regression to evaluate the association between PHDI and NHHR. A smooth curve fitting technique was further employed to investigate possible linear correlations between PHDI and sarcopenia. The subgroup analyses were performed to explore the connection between PHDI and sarcopenia across various demographic groups.

To strengthen the robustness of our findings, we further excluded participants taking lipid-lowering medications and conducted a receiver operating characteristic (ROC) curve analysis to compare the predictive performance of NHHR and protein intake for sarcopenia.

Using the “mediation” package in R, mediation analysis was conducted to assess the indirect, direct, and total effects. To ascertain whether NHHR mediated the association between PHDI and sarcopenia, a bootstrapping approach with 1,000 iterations was utilized. The formula used to calculate the mediation proportion was: indirect effect/(indirect effect + direct effect) × 100% (21). The overall effect of PHDI on sarcopenia (Path C), the direct influence of PHDI on sarcopenia when considering NHHR (Path C′), the impact of PHDI on NHHR (Path A), the influence of NHHR on sarcopenia (Path B), and the indirect effect of NHHR on the relationship between PHDI and sarcopenia (Path A*B) were all communicated as regression coefficients. A *p*-value of less than 0.05 was established as the threshold for statistical significance.

## Results

### Baseline characteristics

A total of 9,094 participants aged 20–85 years were included in this study, representing an estimated 80.05 million U. S. adults. The mean (SD) score for the PHDI among participants was 60.65 (15.28), with a nearly equal distribution of males and females. Among the participants, 788 were diagnosed with sarcopenia, while 8,306 were not. Initial analyses indicated that individuals with sarcopenia were older and had a higher proportion of non-Hispanic Whites, elevated education levels, greater socioeconomic status, a higher prevalence of heavy alcohol consumption, lower PHDI scores, and increased NHHR levels compared to those without sarcopenia. Additional details are provided in Table 1.

**Table 1:** Baseline characteristics of all participants were stratified by Sarcopenia.

Characteristic	Overall, *N* = 9,094 (100%)	Non-sarcopenia, *N* = 8,306 (92.7%)	Sarcopenia, *N* = 788 (7.3%)	*p*-value
Age (%)				**<0.001**
20–40	4,785 (52%)	4,494 (53%)	291 (40%)	
>40	4,309 (48%)	3,812 (47%)	497 (60%)	
Gender (%)				0.181
Male	4,419 (50%)	4,033 (49%)	386 (53%)	
Female	4,675 (50%)	4,273 (51%)	402 (47%)	
Race (%)				**<0.001**
Non-Hispanic White	3,246 (61%)	3,035 (62%)	211 (48%)	
Other	2,594 (17%)	2,346 (16%)	248 (22%)	
Non-Hispanic Black	1,893 (11%)	1,840 (12%)	53 (3.9%)	
Mexican American	1,361 (11%)	1,085 (9.4%)	276 (26%)	
Married/live with partner (%)				0.874
No	3,645 (38%)	3,359 (38%)	286 (39%)	
Yes	5,449 (62%)	4,947 (62%)	502 (61%)	
Education level (%)				**<0.001**
Below high school	1,577 (12%)	1,337 (11%)	240 (22%)	
High School or above	7,517 (88%)	6,969 (89%)	548 (78%)	
PIR (%)				**<0.001**
Not Poor	6,911 (76%)	6,396 (77%)	528 (67%)	
poor	2,183 (24%)	1,910 (23%)	260 (33%)	
Smoking (%)				0.117
Never	5,580 (59%)	5,075 (59%)	505 (61%)	
Former	1,520 (19%)	1,370 (19%)	150 (22%)	
Current	1,994 (21%)	1,861 (22%)	133 (17%)	
Drinking (%)				**<0.001**
Former	728 (8.0%)	648 (7.8%)	102 (13%)	
Heavy	2,455 (27%)	2,243 (27%)	221 (28%)	
Mild	3,092 (34%)	2,907 (35%)	197 (25%)	
Moderate	1,910 (21%)	1,744 (21%)	110 (14%)	
Never	909 (10.0%)	764 (9.2%)	158 (20%)	
Hypertension (%)				**<0.001**
No	6,517 (73%)	6,053 (74%)	464 (58%)	
Yes	2,577 (27%)	2,253 (26%)	324 (42%)	
Diabetes (%)				**<0.001**
No	8,059 (91%)	7,464 (92%)	595 (80%)	
Yes	1,035 (8.8%)	842 (7.9%)	193 (20%)	
High cholesterol (%)				**0.004**
No	6,849 (75%)	6,327 (75%)	522 (68%)	
Yes	2,245 (25%)	1,979 (25%)	266 (32%)	
PHDI [mean (SD)]	60.65 (15.28)	60.81 (15.33)	58.55 (14.56)	**0.013**
PHDI (%)				0.062
T1	3,099 (33%)	2,831 (33%)	268 (37%)	
T2	3,011 (33%)	2,721 (33%)	290 (35%)	
T3	2,984 (33%)	2,754 (34%)	230 (28%)	
NHHR [mean (SD)]	2.93 (1.50)	2.90 (1.48)	3.37 (1.72)	**<0.001**
NHHR (%)				**<0.001**
T1	3,033 (33%)	2,883 (34%)	150 (20%)	
T2	2,977 (33%)	2,720 (33%)	257 (33%)	
T3	3,084 (33%)	2,703 (32%)	381 (48%)	

Mean (SD) for continuous variables: the *P*-value was calculated by the *T*-test.

Percentages (*N*, %) for categorical variables: the *P*-value was calculated by the chi-square test.

PHDI, Planetary Health Diet Index; NHHR, non-high-density lipoprotein cholesterol to high-density lipoprotein cholesterol ratio; PIR, ratio of family income to poverty.

Bolding indicates a *p*-value of less than 0.05.

### Association between PHDI and sarcopenia

**Figure 2:**
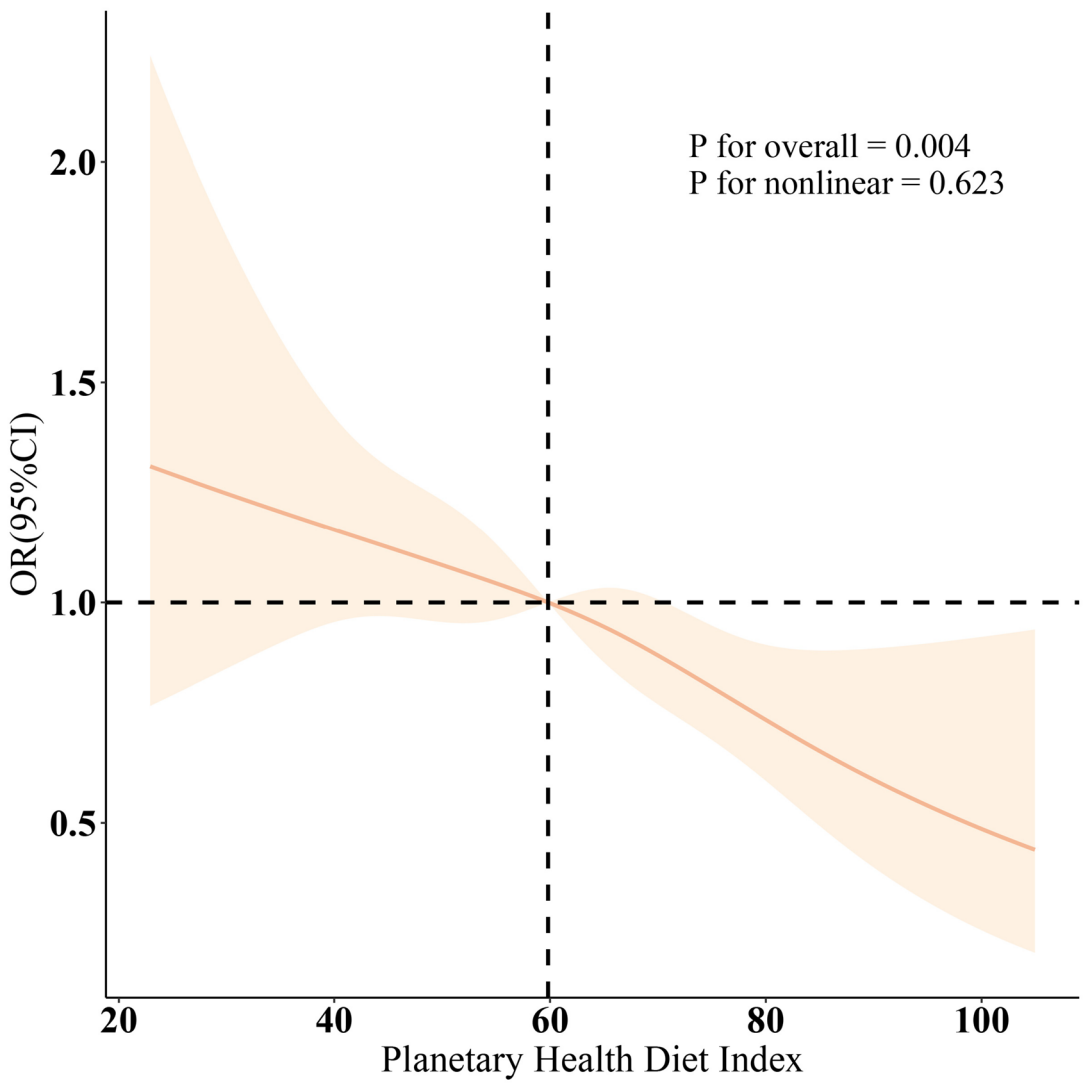
Dose–response relationships between PHDI and Sarcopenia. OR (solid lines) and 95% confidence levels (shaded areas) were adjusted for age, gender, education level, marital, PIR, race, smoking, drinking, hypertension, diabetes, and high cholesterol.

Table 2 illustrates the exploration of the association between PHDI and sarcopenia across three distinct models. In Model 3, following adjustments for all covariates, it was observed that an increase of 10 unit in PHDI was associated with an 14% lower likelihood of sarcopenia (OR = 0.86, 95% CI: 0.79, 0.94). Additionally, individuals in the highest tertile (T3) of PHDI were found to have a 38% reduced likelihood of sarcopenia compared to those in the lowest tertile (T1) (OR = 0.62, 95% CI: 0.43, 0.90). The odds ratios (ORs) indicated a consistent decline across the models as PHDI rose from T1 to T3, with a statistically significant trend being noted (*p* < 0.05). Further validation from the restricted cubic spline (RCS) analysis (Figure 2) suggested the presence of a linear inverse relationship between PHDI and sarcopenia (*p* for non-linearity = 0.623). After excluding participants on lipid-lowering medications, the association between PHDI and sarcopenia remained significant (OR = 0.88, 95% CI: 0.81–0.96), as shown in Supplementary Table S4.

**Table 2:** Association between PHDI, NHHR, and sarcopenia, NHANES 2011–2018.

Characteristics	Model 1 [OR (95% CI)]	*p*-value	Model 2 [OR (95% CI)]	*p*-value	Model 3 [OR (95% CI)]	*p*-value
PHDI – Sarcopenia						
Continuous (per 10-unit)	0.91 (0.85,0.97)	0.005	0.89 (0.83,0.96)	0.002	0.86 (0.79,0.94)	0.001
Tertile						
T1	1 (ref.)		1 (ref.)		1 (ref.)	
T2	0.93 (0.74,1.18)	0.550	0.87 (0.67,1.12)	0.275	0.82 (0.62,1.08)	0.200
T3	0.73 (0.53,0.99)	0.046	0.68 (0.49,0.95)	0.025	0.62 (0.43,0.90)	0.013
*P* for trend	0.046		0.025		0.015	
NHHR – Sarcopenia						
Continuous	1.17 (1.11,1.24)	<0.001	1.12 (1.06,1.18)	<0.001	1.09 (1.03,1.16)	0.007
Tertile						
T1	1 (ref.)		1 (ref.)		1 (ref.)	
T2	1.72 (1.25,2.36)	0.001	1.56 (1.11,2.19)	0.007	1.43 (1.00,2.06)	0.050
T3	2.63 (1.94,3.55)	<0.001	2.15 (1.57,2.96)	<0.001	1.87 (1.36,2.58)	<0.001
*P* for trend	<0.001		<0.001		<0.001	

Model 1: no covariates were adjusted.

Model 2: age, gender, education level, marital, PIR, and race were adjusted.

Model 3: age, gender, education level, marital, PIR, race, smoking, drinking, hypertension, diabetes, total energy intake, protein intake, Urea level, and total serum protein were adjusted.

PHDI, Planetary Health Diet Index; NHHR, non-high-density lipoprotein cholesterol to high-density lipoprotein cholesterol ratio; PIR, ratio of family income to poverty; OR, odds ratio; CI, confidence interval.

### Association between PHDI and NHHR

Following the adjustment for all covariates such as age, gender, education level, marital, PIR, race, smoking, drinking, hypertension, diabetes, total energy intake, protein intake, Urea level, and total serum protein, a notable negative correlation between PHDI and NHHR was observed (*β* = −0.09, 95% CI: −0.11, −0.06, *p* < 0.001), as described in Supplementary Table S3.

### Subgroup analysis of the relationship between PHDI and sarcopenia

**Figure 3:**
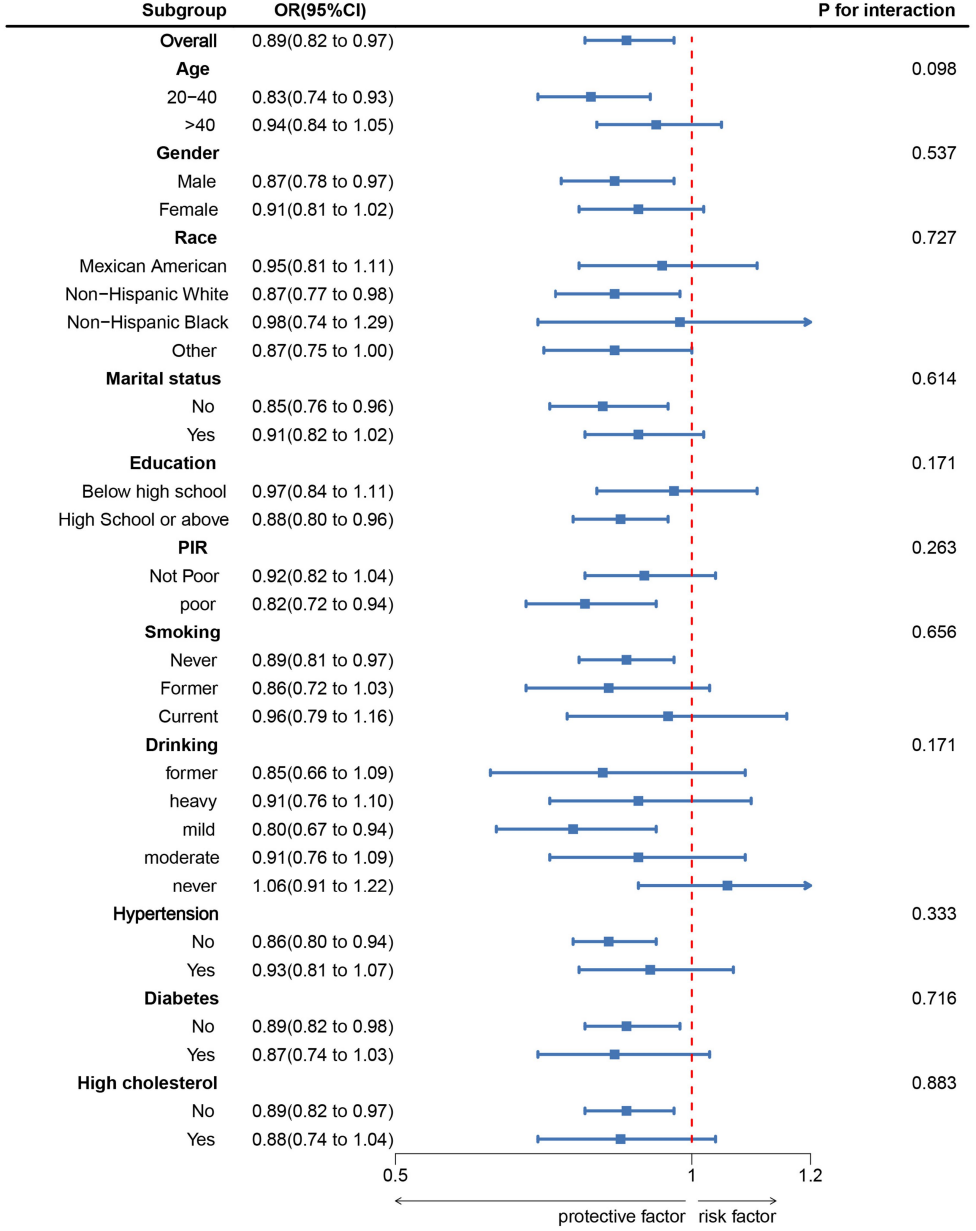
Subgroup analysis between PHDI and Sarcopenia. ORs were calculated as per 10-unit increase in PHDI. Analyses were adjusted for age, gender, education level, marital, PIR, race, smoking, drinking, hypertension, diabetes, and high cholesterol.

The findings of the subgroup analysis examining the association between PHDI and sarcopenia are presented in Figure 3. Stratified analyses were performed considering various covariates such as age, gender, education level, marital status, PIR, race, smoking habits, alcohol consumption, hypertension, diabetes, and high cholesterol levels. No significant interactions were observed between PHDI and any of the stratified factors, indicating that the negative correlation between PHDI and sarcopenia appeared consistent across the different subgroups.

### Association between NHHR and sarcopenia

Table 2 presents the association between NHHR and sarcopenia. In Model 3, following adjustments for all variables, an increase of 10 unit in NHHR was associated with a 9% higher likelihood of sarcopenia (OR = 1.09, 95% CI: 1.03, 1.16). Moreover, individuals in the highest NHHR tertile (T3) were observed to have a 87% higher likelihood of sarcopenia compared to their counterparts in the lowest tertile (T1) (OR = 1.87, 95% CI: 1.36, 2.58). Across the various models, the odds ratios showed a consistent increase as NHHR progressed from T1 to T3, and the trend analysis supported this observed pattern (*p* < 0.001). After excluding participants on lipid-lowering medications, the association between NHHR and sarcopenia remained significant (OR = 1.07, 95% CI: 1.04, 1.11), as shown in Supplementary Table S4.

### Mediation effect

**Figure 4:**
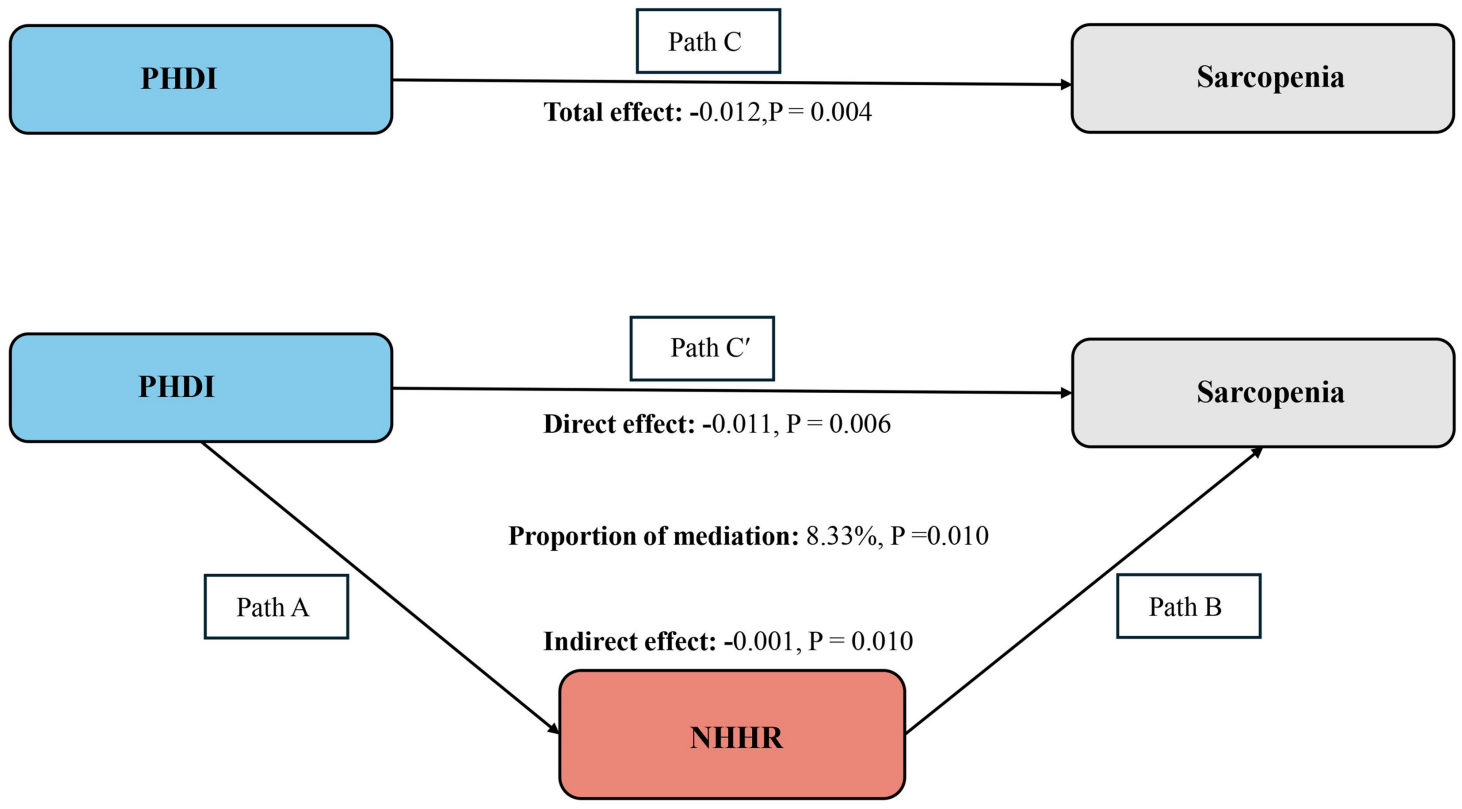
Schematic diagram of the mediation effect analysis. Path C indicates the total effect; path C′ indicates the direct effect. The indirect effect is estimated as the multiplication of paths A and B (path A*B). The mediated proportion is calculated as indirect effect/(indirect effect + direct effect) × 100%. PHDI, Planetary Health Diet Index; NHHR, non-high-density lipoprotein cholesterol to high-density lipoprotein cholesterol ratio. Analyses were adjusted for age, gender, education level, marital, PIR, race, smoking, drinking, hypertension, diabetes, and high cholesterol.

Following the earlier findings, a mediation analysis was performed. After controlling for all covariates (including NHHR), it was determined that NHHR serves as a partial mediator in the association between PHDI and sarcopenia (indirect effect = −0.001, *p* = 0.010; direct effect = −0.011, *p* = 0.006), which accounts for 8.33% of the total effect (mediation proportion = indirect effect/(indirect effect + direct effect) × 100%, p = 0.010). These results suggest that NHHR may function as a mediator in the observed association between PHDI and sarcopenia. The mediation pathways (Path A, Path B, and Path C) along with their effects are depicted in Figure 4.

### ROC curve analysis for sarcopenia

The ROC curve analysis (see Supplementary Figure S1) showed that NHHR had a higher predictive performance for sarcopenia, with an AUC of 0.613 (95% CI: 0.594–0.632), sensitivity of 70.1%, and specificity of 48.2% at a threshold of 2.562. In comparison, protein intake had a lower AUC of 0.552 (95% CI: 0.531–0.573), sensitivity of 63.3%, and specificity of 45.8% at a threshold of 82.523. The difference in AUC was statistically significant (*p* < 0.001), indicating that NIHR is a more reliable predictor of sarcopenia.

## Discussion

This study highlights a significant association between adherence to the PHDI and sarcopenia, with higher PHDI scores associated with a reduced likelihood of sarcopenia. Additionally, our findings suggest that the NHHR may act as a partial mediator in this association. RCS and subgroup analyses further support these findings, demonstrating that increased PHDI adherence is associated with a lower prevalence of sarcopenia.

There is an increasing amount of evidence linking PHDI to the likelihood of several chronic diseases (22, 23). Although there are limited studies directly examining the relationship between PHDI and sarcopenia, current research suggests an association between healthy eating patterns and the maintenance of muscle mass (24). A systematic review of prospective cohort studies indicates that adhering to a diet rich in plant-based foods may reduce the likelihood of developing sarcopenia (25). Additionally, a cross-sectional study involving 12,000 individuals reported that greater intake of whole grains, nuts, and vegetables was associated with a decreased likelihood of sarcopenia (OR: 0.64, 95% CI: 0.49–0.84) (26). Together, these findings support the understanding of the negative association between PHDI and sarcopenia, emphasizing the potential importance of diet quality for maintaining muscle health.

Additionally, emerging studies emphasize the significance of NHHR in relation to sarcopenia. Various cross-sectional investigations have reported a negative correlation between the ratio of triglycerides to HDL cholesterol and relative grip strength, suggesting that markers of lipid metabolism may serve as predictors of muscle degeneration (27). Furthermore, there is evidence indicating that fatty acids and muscle metabolism are interconnected processes contributing to muscle loss (28). For example, research involving 9,012 participants identified an association between NHHR and an increased likelihood of reduced muscle mass in middle-aged Americans (13). Consistent with these findings, our study shows a positive association between NHHR and sarcopenia, highlighting NHHR’s potential as an indicator of muscle deterioration. This underscores the importance of NHHR for understanding the fundamental mechanisms underlying sarcopenia.

This study examines the association between the PHDI and sarcopenia, identifying the NHHR as a potential mediator. Both inflammation and lipid metabolism are known to contribute to the likelihood of sarcopenia (29, 30), with elevated pro-inflammatory markers, such as Tumor Necrosis Factor-alpha (TNF-*α*), Interleukin-6(IL-6), and C-Reactive Protein (CRP), being observed in both sarcopenia and dyslipidemia, suggesting shared pathogenic mechanisms (31–33). Inflammatory mediators may influence lipid metabolism by stimulating lipolysis in adipocytes, increasing free fatty acid (FFA) levels, and promoting insulin resistance (34, 35). Such metabolic disturbances are linked to systemic inflammation, which can exacerbate muscle loss. Elevated FFAs and Low-Density Lipoprotein (LDL) may further trigger oxidative stress and inflammation, impairing muscle function and regeneration (36, 37). Lipid peroxidation leads to oxidative damage, contributing to muscle fiber deterioration and accelerating sarcopenia (38). Additionally, recent studies have highlighted the broader implications of lipid metabolism in related health outcomes. For example, dysregulated lipid metabolism has been linked to the progression of liver steatosis, where malnutrition and impaired hepatic metabolism can negatively impact overall health (39). Moreover, emerging evidence suggests that HDL-bound non-coding RNAs play a regulatory role in cardiovascular risk, offering insights into the molecular interactions between lipid metabolism and systemic inflammation (40). Given the shared inflammatory and metabolic pathways between cardiovascular disease and sarcopenia, these novel biomarkers may also have relevance in predicting muscle health and function. Dietary patterns play a crucial role in modulating inflammation and lipid metabolism, thereby affecting the likelihood of sarcopenia (41). The PHDI, which emphasizes plant-based food intake, aligns with these protective effects. For example, polyphenols found in fruits and vegetables possess anti-inflammatory properties, contributing to lower systemic inflammation (42). Additionally, a diet rich in fiber and antioxidants from whole grains can improve lipid metabolism by reducing LDL and FFA levels (43). Previous research supports that plant-based diets can modulate lipid profiles and inflammation, which are essential for muscle preservation (44–46). In our study, NHHR acted as a mediator in the PHDI-sarcopenia relationship, reinforcing the importance of diet quality for maintaining muscle health. Higher PHDI scores are associated with better lipid profiles and lower inflammation, both of which are protective against sarcopenia.

The ROC curve analysis indicated that NHHR outperformed protein intake in predicting sarcopenia, with a significantly higher AUC. These results indicate the potential utility of NHHR may as a more reliable biomarker for mediating sarcopenia assessment.

Even after excluding participants on lipid-lowering medications, the associations of both PHDI and NHHR with sarcopenia remained significant. These findings highlight the robustness of the relationships and suggest that both dietary patterns and lipid metabolism play critical roles in the pathogenesis of sarcopenia.

Our study highlights the potential clinical use of PHDI and NHHR scores in identifying and managing patients at sarcopenia. Both scores correlate with sarcopenia likelihood, making them useful tools for clinicians. Integrating these scores into clinical practice can help identify patients who may benefit from preventive interventions, such as personalized dietary advice and tailored exercise programs to address lipid imbalance and muscle loss. Regular monitoring of these scores could also track sarcopenia progression and guide treatment adjustments. Although further validation is needed, incorporating PHDI and NHHR scores into routine assessments could improve sarcopenia prevention and management in at-risk populations.

This study has several strengths. First, the PHDI serves as a comprehensive metric of both healthy eating and environmental sustainability, demonstrating its potential to predict sarcopenia. By promoting plant-based food intake while limiting red meat and processed foods, PHDI is closely associated with improvements in muscle health. Second, our analysis utilized a large, nationally representative dataset, enhancing the generalizability of our findings. Finally, to the best of our knowledge, this study is the first to explore NHHR as a mediator between PHDI and sarcopenia, addressing a significant research gap.

However, this study has several limitations. First, the cross-sectional design precludes establishing causality between PHDI, NHHR, and sarcopenia. While the mediation analysis suggests a potential pathway involving NHHR, prospective cohort studies, randomized controlled trials, or experimental research are necessary to confirm these findings and elucidate the causal mechanisms. Second, NHANES relies on self-reported data, including dietary intake via food frequency questionnaires and clinical conditions, which may introduce recall bias or misclassification. Self-reported data inherently carry the risk of bias and should be interpreted with caution. Using more objective measures, such as physician-confirmed diagnoses or dietary biomarkers, could improve result reliability. Third, despite adjusting for numerous potential confounders, residual confounding cannot be entirely ruled out due to the inherent limitations of the NHANES dataset. Factors like genetic predispositions, environmental exposures, or unmeasured lifestyle variables may influence the observed associations. Lastly, while NHANES data are nationally representative of the U. S. population, the generalizability of these findings to other populations with differing dietary habits, demographics, or health profiles may be limited. Future longitudinal and experimental studies are warranted to validate these results and explore the mechanisms in greater depth.

## Conclusion

In conclusion, elevated PHDI levels are associated with a lower likelihood of sarcopenia, whereas diminished NHHR levels might enhance this connection. Therefore, following a PHDI-oriented dietary plan and managing NHHR levels may hold substantial clinical importance in reducing the likelihood of sarcopenia.

## Data Availability

Publicly available datasets were analyzed in this study. This data can be found at: https://www.cdc.gov/nchs/nhanes/.
