# Supplementary material for: Linking the Planetary Health Diet Index to sarcopenia: the mediating effect of the non-high-density lipoprotein cholesterol to high-density lipoprotein cholesterol ratio (NHHR)
Source: Front Nutr. 2025 Jun 19;12:1504037. doi: 10.3389/fnut.2025.1504037 (PMC12222211; doi:10.3389/fnut.2025.1504037)
Supplement: Supplementary file 1 [file Table_1.DOCX]

**Supplementary Material**

| **Table S1.** Scoring criteria for the Planetary Health Diet Index (PHDI). | | |
| --- | --- | --- |
| **Dietary component** | **Category minimum score** (0 points) | **Category maximum score** (10 points) |
| ***Adequacy components*** | | |
| Whole grains^1^ | 0 grams | ≥ 75 grams for women  ≥ 90 grams for men |
| Whole fruits (excludes fruit juice) | 0 grams | ≥ 200 grams |
| Non-starchy vegetables | 0 grams | ≥ 300 grams |
| Nuts and seeds | 0 grams | ≥ 50 grams |
| Legumes |  |  |
| Non-soy legumes^2,3^ | 0 grams | 100 grams |
| Soybean/ soy foods^2,3^ | 0 grams | 50 grams |
| Unsaturated oils | 0% of total energy intake | ≥ 10% of total energy intake |
| ***Moderation components*** | | |
| Starchy vegetables | ≥ 200 grams | ≤ 50 grams |
| Dairy^4^ | ≥ 4.08 cup-equivalents | ≤ 1.02 cup-equivalents |
| Red and processed meat | ≥ 300 grams | ≤ 14 grams |
| Poultry | ≥ 58 grams | ≤ 29 grams |
| Eggs | ≥ 120 grams | ≤ 12 grams |
| Fish | ≥ 50 grams | ≤ 15 grams |
| Saturated oils and *trans* fats | ≥ 21% of total energy intake | ≤ 3.5% of total energy intake |
| Added sugar and fruit juice | ≥ 25% of total energy intake | ≤ 5% of total energy intake |
| ^1^Thresholds were based on the midpoint of the recommended range listed in the EAT-Lancet Commission Scientific Report.  ^2^Grams per day calculated from dry weight.  ^3^To calculate the score for the legumes component, the nonsoy and soy subcomponents were each weighted at 0.5.  ^4^In FPED, 1 serving of dairy is equal to 245 g of whole-milk or derivative equivalent. In the EAT-Lancet report, scores were assigned ≤250 g whole-milk or derivative equivalent for the maximum score or ≥1000 g whole-milk or derivative equivalent for the minimum score.  Note: The above dietary components were obtained from the Food Pattern Equivalence Database (FPED) of the 24-hour dietary recall data(https://www.ars.usda.gov/northeast-area/beltsville-md-bhnrc/beltsville-human-nutrition-research-center/food-surveys-research-group/docs/fped-databases/). | | |

**Table S2.** Description of covariates.

| Covariates | Description in NHANES |
| --- | --- |
| Age | Divided into two groups: 20-40 years old and >40 years old |
| Gender | Male and Female |
| Race | Non-Hispanic White,Non-Hispanic Black,Mexican American , Other Race |
| Marital status | Yes: Married/Living with partner;No |
| Educational level | Below high school, High School or above |
| PIR | Poor: <1.3; Not Poor:>=1.3 |
| Smoking | Smoking status was grouped into never smoker (defined as <100 cigarettes in a lifetime), former smoker (defined as ≥100 cigarettes and had quit smoking), and current smoker (defined as ≥100 cigarettes in a lifetime) |
| Drinking | heavy drinking (≥4 drinks/day for men, ≥3 drinks/day for women, or ≥5 days of drinking in a month),  moderate drinking (≥3 drinks/day for men, ≥2 drinks/day for women, or ≥2 days of drinking in a month),  mild drinking (≤2 drinks/day for men, ≤1 drink/day for women, and ≥12 drinks in a year),  and never-drinking (total number of drinks in a year <12, and dietary alcohol content of 0%) |
| Hypertension | The diagnostic criteria consist of self-reported hypertension history, the utilization of antihypertensive medication, a systolic blood pressure (SBP) ≥ 140mmHg, or a diastolic blood pressure (DBP) ≥ 90mmHg |
| Diabetes | Diabetes was defined as a history of previous diabetes, HbA1c level ≥6.5%, or fasting blood glucose level ≥126 mg/dL |
| High cholesterol | Participants were asked whether they had high cholesterol |

PIR, Ratio of family income to poverty.

**Table S3.** Multivariate linear regression of PHDI and NHHR.

|  | β | 95%CI | P-value |
| --- | --- | --- | --- |
| PHDI - NHHR | -0.09 | (-0.11, -0.06) | <0.001 |

Adjusted for age, gender, education level, marital, PIR, race, smoking, drinking, hypertension, diabetes, total energy intake, protein intake, Urea level, and total serum protein.

**Table S4.** Association between PHDI, NHHR, and Sarcopenia, excluding participants taking lipid-lowering medications.

| **Characteristics** | **Model 1 [OR (95% CI)]** | ***p-value*** | **Model 2 [OR (95% CI)]** | ***p-value*** | **Model 3 [OR (95% CI)]** | ***p-value*** |
| --- | --- | --- | --- | --- | --- | --- |
| **PHDI - Sarcopenia** |  |  |  |  |  |  |
| Continuous (per 10 scores) | 0.88(0.82,0.96) | 0.003 | 0.88(0.81,0.96) | 0.004 | 0.88(0.81,0.96) | 0.005 |
| Tertile |  |  |  |  |  |  |
| T1 | 1 (ref.) |  | 1 (ref.) |  | 1 (ref.) |  |
| T2 | 0.99(0.77,1.28) | 0.970 | 0.98(0.74,1.30) | 0.900 | 0.97(0.71,1.34) | 0.870 |
| T3 | 0.74(0.53,0.99) | 0.042 | 0.74(0.51,0.97) | 0.032 | 0.74(0.52,0.98) | 0.042 |
| *P for trend* | 0.043 |  | 0.030 |  | 0.040 |  |
| **NHHR - Sarcopenia** |  |  |  |  |  |  |
| Continuous | 1.15(1.05,1.26) | 0.003 | 1.10(1.02,1.20) | 0.010 | 1.07(1.04,1.11) | 0.030 |
| Tertile |  |  |  |  |  |  |
| T1 | 1 (ref.) |  | 1 (ref.) |  | 1 (ref.) |  |
| T2 | 1.57(1.03,2.38) | 0.030 | 1.42(0.90,2.22) | 0.130 | 1.37(0.84,2.23) | 0.200 |
| T3 | 2.19(1.36,3.52) | 0.002 | 1.77(1.05,2.99) | 0.030 | 1.73(1.00,3.01) | 0.050 |
| *P for trend* | 0.001 |  | 0.030 |  | 0.050 |  |

Model 1: no covariates were adjusted.

Model 2: age, gender, education level, marital, PIR, and race were adjusted.

Model 3: age, gender, education level, marital, PIR, race, smoking, drinking, hypertension, diabetes, total energy intake, protein intake, Urea level, and total serum protein were adjusted.

Abbreviation: PHDI, Planetary Health Diet Index; NHHR, Non-high-density lipoprotein cholesterol to high-density lipoprotein cholesterol ratio; PIR, Ratio of family income to poverty; OR, odds ratio; CI, confidence interval.


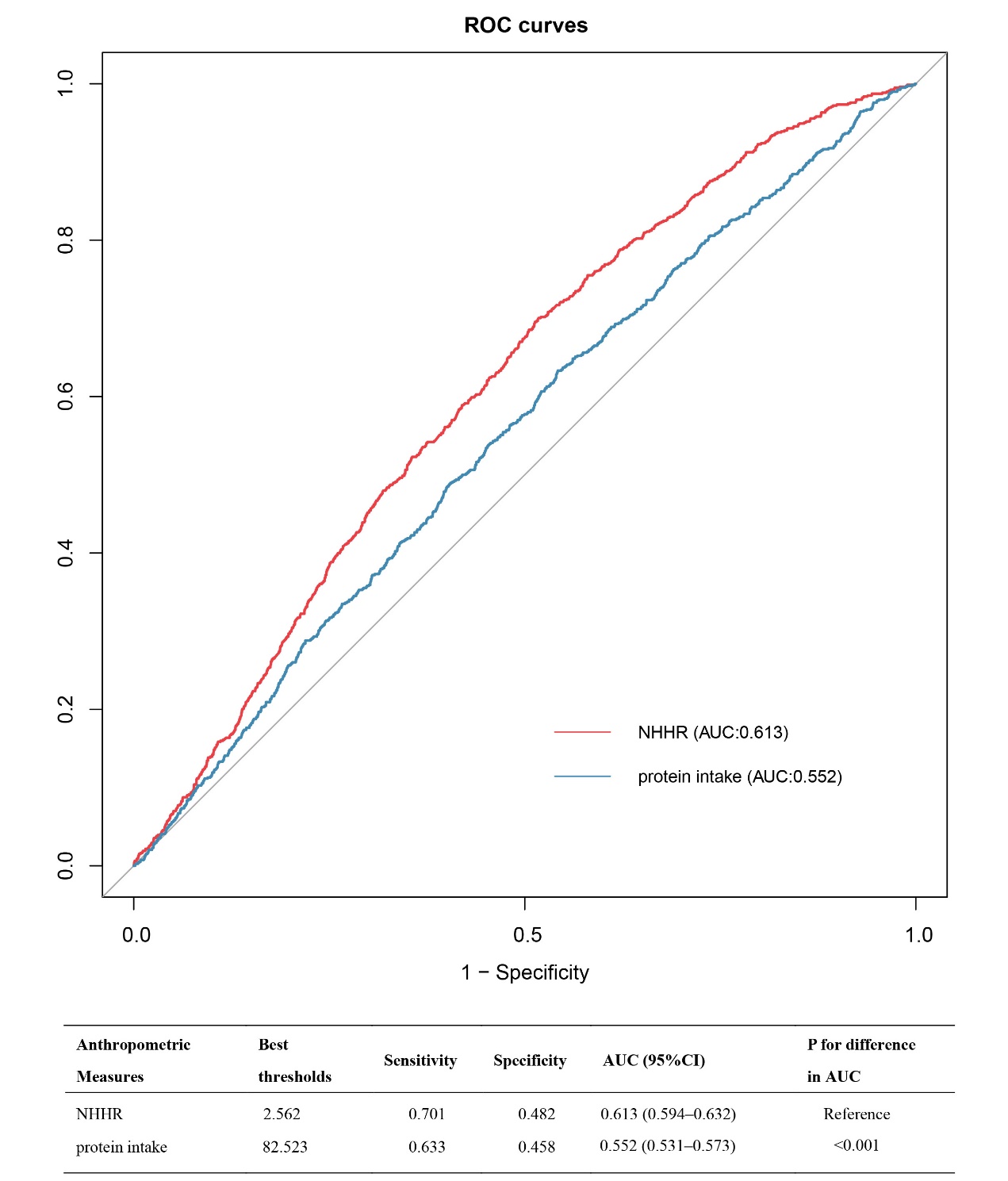


**Figure S1.** Receiver operating characteristic (ROC) curve analysis for Sarcopenia.
